# Crosstalk between macrophages and fibroblasts contributes to inflammation and damage in giant cell arteritis

**DOI:** 10.1093/rheumatology/keaf408

**Published:** 2025-08-07

**Authors:** Shuang Xu, William F Jiemy, Anqi Zhang, Fokke Walinga, Miranda Nijenhuis, Elien Hensema, Wayel Abdulahad, Kornelis SM van der Geest, Peter Heeringa, Annemieke Boots, Elisabeth Brouwer, Maria Sandovici

**Affiliations:** Department of Rheumatology and Clinical Immunology, University Medical Center Groningen, University of Groningen, Groningen, the Netherlands; Department of Rheumatology and Clinical Immunology, University Medical Center Groningen, University of Groningen, Groningen, the Netherlands; Department of Rheumatology and Clinical Immunology, University Medical Center Groningen, University of Groningen, Groningen, the Netherlands; Department of Pathology and Medical Biology, University Medical Center Groningen, University of Groningen, Groningen, the Netherlands; Department of Dermatology, University Medical Center Groningen, University of Groningen, Groningen, the Netherlands; Department of Rheumatology and Clinical Immunology, University Medical Center Groningen, University of Groningen, Groningen, the Netherlands; Department of Rheumatology and Clinical Immunology, University Medical Center Groningen, University of Groningen, Groningen, the Netherlands; Department of Rheumatology and Clinical Immunology, University Medical Center Groningen, University of Groningen, Groningen, the Netherlands; Department of Pathology and Medical Biology, University Medical Center Groningen, University of Groningen, Groningen, the Netherlands; Department of Rheumatology and Clinical Immunology, University Medical Center Groningen, University of Groningen, Groningen, the Netherlands; Department of Rheumatology and Clinical Immunology, University Medical Center Groningen, University of Groningen, Groningen, the Netherlands; Department of Rheumatology and Clinical Immunology, University Medical Center Groningen, University of Groningen, Groningen, the Netherlands

**Keywords:** giant cell arteritis, macrophages, fibroblasts, inflammation, matrix metalloproteinases, tenascin-C

## Abstract

**Objectives:**

Giant cell arteritis (GCA) is a large vessel vasculitis characterized by arterial wall inflammation and remodelling. Macrophages and fibroblasts are abundantly present in arteries affected by GCA, but their crosstalk in GCA pathogenesis is largely unknown. Here we investigated the interaction between macrophages and fibroblasts in GCA-affected arteries and *in vitro.*

**Methods:**

Immunostaining was performed to detect macrophages (CD68, CD206, FRβ), fibroblasts (CD90, CD200), GM-CSF, M-CSF, IL-6, MMP-3 and tenascin-C in GCA-positive temporal arteries (*n* = 9) and aorta tissues (*n* = 9). Serum tenascin-C levels were measured by ELISA in GCA patients (*n* = 36) and healthy controls (*n* = 46). *In vitro*, monocytes isolated from peripheral blood mononuclear cells of healthy donors (*n* = 10) were incubated with GM-CSF or M-CSF for 8 days to induce macrophage differentiation. GM-CSF/M-CSF-macrophage-conditioned medium (MCM) was added to human aortic adventitial fibroblast (HAoAF) cultures for 24 h. mRNA expression of proinflammatory cytokines(IL-6, IL-1β), growth factors (GM-CSF, M-CSF), matrix metalloproteinase (MMP-1, MMP-3), chemokines (CCL2, CX3CL1), extracellular matrix proteins (Col1a1, Col1a2, Col3a1, fibronectin-1, tenascin-C) and phenotypic markers (fibroblast activation protein [FAP], podoplanin [PDPN], α-smooth muscle actin, CD200) in cultured fibroblasts were examined by qPCR.

**Results:**

In GCA-affected arteries, pro-inflammatory CD90^+^IL-6^+^ fibroblasts, but not pro-resolving CD90^+^CD200^+^ fibroblasts, were spatially associated with macrophages. Adventitial CD90^+^ fibroblasts expressed GM-CSF and/or M-CSF, which linked to macrophage subset distribution. *In vitro*, both GM-CSF- and, to a lesser extent, M-CSF-derived MCM upregulated mRNA expression of IL-6, GM-CSF, M-CSF, CCL2, PDPN and CD200 in fibroblasts. Upregulation of IL-1β, MMP-3, Col3a1 and tenascin-C and downregulation of FAP in fibroblasts was observed with GM-CSF-derived MCM. Adventitial CD90^+^ fibroblasts in GCA-affected temporal arteries also expressed MMP-3 and tenascin-C. Serum levels of tenascin-C in patients with treatment-naïve GCA were significantly higher than those in healthy controls, showing a good diagnostic accuracy (area under the curve 0.89).

**Conclusion:**

The interaction between fibroblasts and macrophages may contribute to the chronicity and progression of GCA and deserves further investigation. Serum tenascin-C is a candidate biomarker for GCA diagnosis.

## Graphical Abstract

**Figure:**
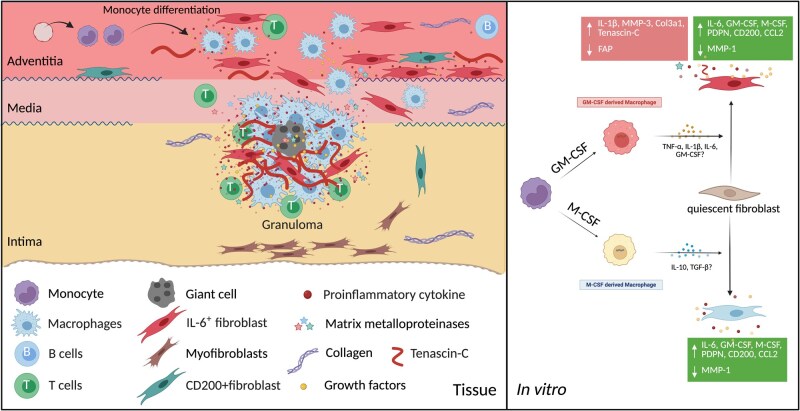

Rheumatology key messages

- In GCA-affected arteries, pro-inflammatory CD90^+^IL-6^+^ fibroblasts but not pro-resolving CD90^+^CD200^+^ fibroblasts are spatially associated with macrophages.
- Soluble factors derived from GM-CSF-induced macrophages and, to a lesser extent, M-CSF-induced macrophages skew human aortic adventitial fibroblasts towards a pro-inflammatory and pro-destructive phenotype *in vitro*.
- Tenascin-C, an extracellular matrix protein mainly expressed by adventitial fibroblasts, should be further investigated as a potential novel biomarker for GCA diagnosis.

## Introduction

Giant cell arteritis (GCA) is the most frequent form of large vessel vasculitis in individuals over 50 [1, 2]. It affects medium to large arteries and presents as a spectrum of cranial GCA (C-GCA) and large-vessel GCA (LV-GCA) [3, 4]. C-GCA mainly affects the extracranial branches of the carotid artery, including the temporal artery, whereas LV-GCA mostly affects the aorta and its major branches. The most feared complications of GCA, being visual loss and stroke in C-GCA and the rupture of an aortic aneurysm in LV-GCA, are the consequence of chronic arterial inflammation and pathological remodelling [5, 6]. While glucocorticoids and the IL-6 receptor (IL-6R) blocker tocilizumab are largely effective in suppressing systemic inflammation, the relapse rate remains high and their effect on arterial inflammation and damage is still debated [7]. Understanding the exact mechanisms of chronic arterial inflammation and remodelling in GCA may open up new avenues for effective therapeutic intervention in GCA. While the role of infiltrating immune cells such as macrophages and T cells has been extensively studied, the involvement of resident vascular cells such as the fibroblasts in GCA is less well defined [8]. Macrophages and fibroblasts interact with each other at the sites of inflammation in other chronic inflammatory diseases [9, 10].

Macrophages play a crucial role in GCA, which is reflected not only by their abundance in arterial lesions but also by their well-known role in inflammation and vascular damage. Our group previously identified the spatially distinct distribution of two macrophage subsets in GCA lesions. While CD206^+^ macrophages were mainly found in the media and highly associated with inflammation (i.e. release of IL-6, IL-1β, TNF-α) and tissue destruction (MMP-9), folate receptor beta (FRβ)^+^ macrophages were mainly located in the intima and adventitia, and were found to be linked to tissue remodelling and intimal hyperplasia [11]. Local production of a gradient of GM-CSF and M-CSF is suggested to be responsible for the skewing of CD206^+^ and FRβ^+^ macrophages in GCA lesions, respectively [11].

Fibroblasts are the most dominant cell type in the adventitia of the large- and medium-size arteries [12]. Under normal conditions, the primary function of tissue fibroblasts is homeostasis of the connective tissue and extracellular matrix (ECM). However, this function is affected in pathological states, where there is a disbalance between pro-inflammatory/destructive and pro-resolving/healing fibroblasts [13]. Recently, we documented abundant pro-inflammatory IL-6^+^FAP^+^CD90^+^, and destructive MMP-9^+^FAP^+^CD90^+^ fibroblast subpopulations in GCA-affected arteries [14]. Moreover, a pro-resolving subpopulation of CD200^+^ fibroblasts was described in arthritis [15]. It is well established that fibroblasts react to cues from their microenvironment. Macrophages produce large amounts of cytokines, such as TGF-β and TNF-α. TGF-β is related to fibroblast activation and differentiation, and can induce the expression of IL-6 and the macrophage chemoattractant CCL-2 in fibroblasts [16, 17]. TNF-α upregulated IL-6 mRNA expression in human aortic fibroblasts [18].

Tenascin-C is a large hexametric protein of the ECM that exhibits limited expression in healthy tissues but is upregulated in response to tissue damage. Elevated expression of tenascin-C is often observed at sites of inflammation and remodelling in other diseases, for example, in rheumatoid arthritis (RA) [19] and systemic sclerosis (SSc) [20]. Serum tenascin-C levels correlate with joint erosion in patients with RA, and higher joint tenascin-C expression predicts RA development [21, 22]. However, the modulation of tenascin-C and its relevance in GCA are as yet unexplored.

In this study, we sought to investigate macrophage–fibroblast interaction both in vascular tissues and *in vitro.* To this end, we analysed the distribution of fibroblast and macrophage subpopulations in GCA-affected temporal artery biopsy (TAB) and aorta tissues and their spatial relationship with the expression of GM-CSF, M-CSF and connective tissue-related proteins. For *in vitro* studies, we investigated the impact of GM-CSF- and M-CSF-derived macrophage conditioned medium on phenotypic and functional changes in primary human aortic adventitial fibroblasts. In addition, we measured serum tenascin-C levels in a cohort of patients with GCA and healthy controls.

## Methods

### Study population

This study was performed within the GCA/PMR/SENEX (GPS) cohort, which is a prospective longitudinal observational cohort [23]. All patients were seen at the Rheumatology and Clinical Immunology outpatient clinic of the University Medical Centre Groningen (UMCG) between 2011 and 2023. All patients and healthy controls provided signed informed consent prior to inclusion in the GPS cohort. Medical Ethical approval was obtained from the Internal Review Board (study number NL3173404210, METc2010/222 for GCA and SENEX). For use of the aorta tissue, consent from the Internal Review Board and written patient consent were not required under the Dutch law for human medical research (WMO) since this is a retrospective, non-interventional study. Patients were informed that their medical data or tissues could be used for research purposes in accordance with privacy rules (UMCG Research Register Number: 201800370). All the procedures complied with the principles of the Declaration of Helsinki.

For tissue staining, inflamed TAB samples from patients with histologically proven GCA (*n* = 9), non-inflamed TAB samples (*n* = 9, four from patients with PMR, five from non-GCA/non-PMR patients undergoing a TAB due to suspicion of GCA), and aortic tissues from patients with GCA-related (*n* = 9) or atherosclerosis-related (*n* = 11) aneurysms, who underwent aortic aneurysm surgery, were studied. To investigate the impact of macrophage-derived soluble factors on vascular adventitial fibroblasts *in vitro*, peripheral blood mononuclear cells (PBMC) from 10 healthy donors older than 50 years were used. To determine circulating tenascin-C levels, serum samples from GCA patients (*n* = 36) and age- and sex-matched healthy controls (*n* = 46) were included. Patient and healthy control characteristics are shown in Table 1.

**Table 1.** Patient and control characteristics

Tissue analysis	TAB-GCA	TAB control	Aorta-GCA	Aorta-AS
No. of patients	9	9	9	11
Age, median (range), years	72 (59–81)	76 (52–86)	67 (55–79)	65 (59–78)
Sex, female, %	88.9%	55.6%	66.7%	63.6%
No. of subjects on treatment with glucocorticoids/immunosuppression	2	2	0	0
ESR, median (range), mm/h	46 (9–104)	52 (14–74)	24 (4–37)	17 (1–40)
CRP, median (range), mg/l	22 (4–158)	29 (4–87)	5 (1.4–52)	9 (3–32)

ELISA and *in vitro*	Serum-GCA	Serum-HC	PBMC-HC
Number of patients	36	46	10
Age, median (range), years	69.5 (43–92)	68 (51–91)	72 (58–85)
Sex, female, %	69.4%	73.9%	70%
Fulfilled ACR criteria for GCA, *n* (%)	29 (80.6%)	n.a	n.a.
GCA symptoms (C-GCA/LV-GCA/combined), *n*	9/11/16	n.a.	n.a.
Ischemic ocular involvement, *n* (%)	2 (5.6%)	n.a.	n.a.
Claudication, *n* (%)	18 (50%)	n.a.	n.a.
PMR symptoms, *n* (%)	15 (41.7%)	n.a.	n.a.

AS: atherosclerosis; GCA: giant cell arteritis; HC: healthy controls; n.a.: not applicable; PBMC: peripheral blood mononuclear cells; PMR: polymyalgia rheumatica; TAB: temporal artery biopsy.

### Analytical methods

For immunohistochemistry staining, immunofluorescence staining, Opal staining, PBMC isolation, monocyte isolation, macrophage differentiation, fibroblast culture, real-time qPCR, ELISA and Luminex, see Supplementary Data S1, Supplementary Table S1–S4 and Supplementary Fig. S1.

### Statistics

For studies on tissue, the Mann–Whitney *U*-test was used to compare GCA and control in each layer, and to evaluate the association of tissue MMP-3 expression with intima hyperplasia. The correlation between tenascin-C/MMP-3 positivity and the adventitial/intimal thickness score was analysed using Spearman’s test. For the *in vitro* study, Friedman’s test was performed to compare the mRNA expression of various markers in fibroblasts between multiple groups. If significant, the Wilcoxon test was performed to test for differences between two groups. Differences in cytokine production in GM-CSF and M-CSF macrophage supernatants were analysed using the Wilcoxon test. For serum tenascin-C levels, the Mann–Whitney *U*-test was used to compare two groups (unpaired samples: GCA *vs* HC). Samples from different time points of the same patient (paired samples) were analysed using the Wilcoxon signed-rank test. Spearman’s rank correlation coefficient was used to assess the relationship between tenascin-C and ESR, CRP, fibroblast activation protein (FAP), monocyte count and age. A receiver operating characteristic (ROC) curve was constructed using logistic regression to further assess the diagnostic performance of tenascin-C, and the area under the curve (AUC) was calculated to quantify the overall discrimination ability of the logistic regression model. Statistical analyses were performed using GraphPad Prism 9.0 software (GraphPad Software, Boston, MA, USA) and *P* < 0.05 (two-tailed) was considered statistically significant.

## Results

### Pro-inflammatory CD90^+^IL-6^+^ fibroblasts, rather than pro-resolving CD90^+^CD200^+^ fibroblasts, are spatially associated with macrophages in GCA-affected arteries

**Figure 1.**
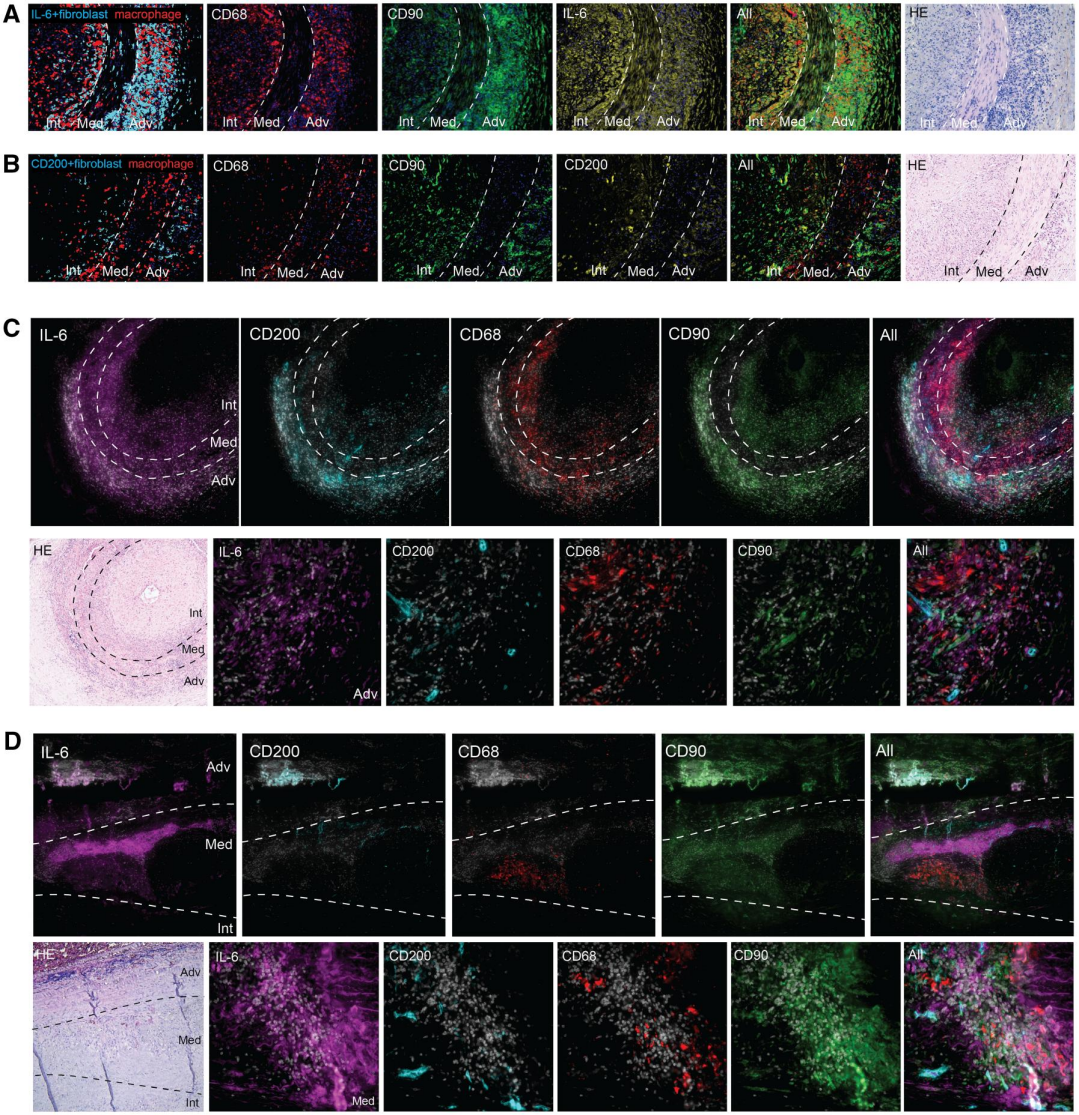
Spatial distribution of fibroblast subtypes (pro-inflammatory CD90^+^IL-6^+^/pro-resolving CD90^+^CD200^+^) and macrophages (CD68^+^) in GCA-affected arterial tissues. (**A**, **B**) Representative images of Opal staining of CD68 (red), CD90 (green) and IL-6 (yellow) and of CD68, CD90 and CD200 (yellow) in GCA-affected temporal artery biopsy (TAB). Overlap of CD90 and IL-6, CD90 and CD200 is shown in cyan; nuclei–4′,6-diamidino-2-phenylindole (DAPI) are shown in blue. (**C**, **D**) Representative four-colour Opal images of CD68, CD90, CD200 and IL-6 in TAB (**C**) and aortic tissues (**D**). The upper panel shows an overview of the vessel, the lower panel shows typical GCA lesions at higher magnification. Nuclei–DAPI are shown in grey. The dotted lines delineate the three layers of the blood vessel: adventitia (Adv), media (Med) and intima (Int). Histology details of the Opal-stained artery sections are shown in haematoxylin and eosin (HE) staining

In GCA-affected TAB, fibroblasts (CD90^+^) were localized predominantly in adventitia and intima, while macrophages (CD68^+^) were localized in all three layers (adventitia, media and intima). In GCA-affected aorta tissues, CD90^+^ fibroblasts were present mainly in the adventitia and media, whereas macrophages were present in the media (Supplementary Fig. S2A–C). CD200 is a marker related to disease resolution in RA [15]. We observed limited CD200 expression in GCA TAB and medial granulomas of aorta tissues (Supplementary Fig. S2D). To address the distribution of fibroblast subpopulations (pro-inflammatory CD90^+^IL-6^+^ fibroblasts and pro-resolving CD90^+^CD200^+^ fibroblasts) at the site of inflammation in GCA, Opal staining of CD90, CD68, CD200 and IL-6 in GCA-affected TABs (*n* = 9) and aorta tissues (*n* = 9) was performed. We found that CD90^+^IL-6^+^ fibroblasts were located predominantly adjacent to macrophages, whereas CD90^+^CD200^+^ fibroblasts were generally located more distant from macrophages (Fig. 1A–D, Supplementary Fig. S2E). CD90^+^IL-6^+^ fibroblasts were more abundant at sites of inflammation than CD90^+^CD200^+^ fibroblasts (Fig. 1A and B).

### Fibroblasts are important sources of GM-CSF or M-CSF and partly associate with CD206^+^ and FRβ^+^ macrophage subset distribution in GCA lesions

**Figure 2.**
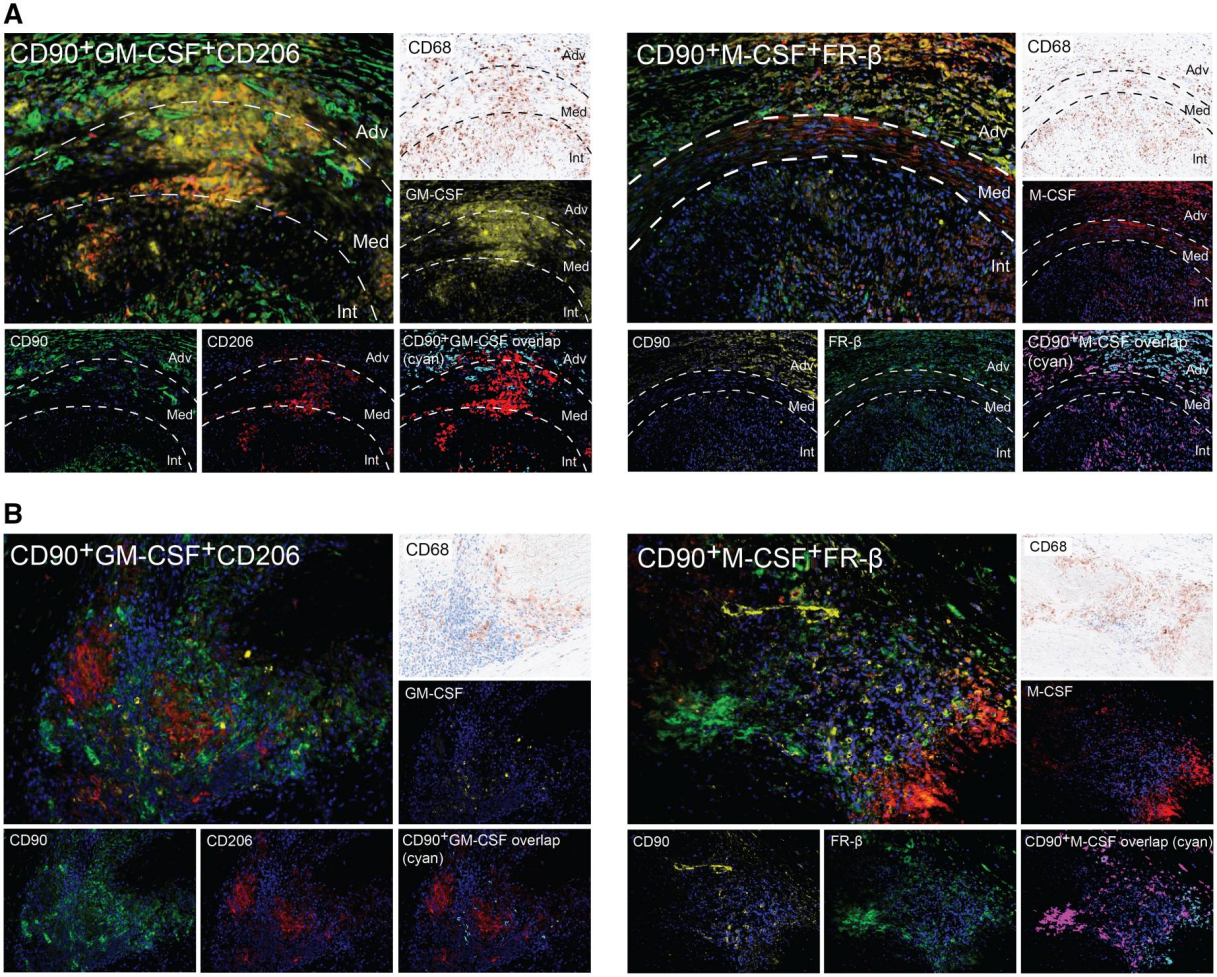
Fibroblast-expressing GM-CSF/M-CSF and their spatial relation to macrophage subsets (CD206^+^ and FRβ^+^) in GCA lesions. (**A**) Representative image from GCA-affected temporal artery biopsy (TAB) showing triple immunofluorescence staining of CD90 (green), GM-CSF (yellow) and CD206 (red) and of CD90 (yellow), M-CSF (red) and FRβ (green). The last image of each pattern shows the colocalization of CD90 and GM-CSF or CD90 and M-CSF (cyan). (**B**) Representative image of GCA-affected aorta tissues showing triple immunofluorescence staining of CD90 (green), GM-CSF (yellow) and CD206 (red) and of CD90 (yellow), M-CSF (red) and FRβ (green). Colocalization of CD90–GM-CSF or CD90–M-CSF is shown in cyan, and is located in the medial granuloma. CD68 shows macrophage immunohistochemistry in brown. Adv: adventitia; Int: intima; Med: media; FRβ: folate receptor β

To determine whether fibroblasts express GM-CSF/M-CSF and associate with the respective macrophage subsets in GCA lesions, triple immunofluorescence (IF) staining for CD90, CD206 and GM-CSF and CD90, FRβ and M-CSF was performed. The colocalization of CD90 and GM-CSF and CD90 and M-CSF was mainly found in the adventitia where CD90^+^ cells contributed to 55% of cellular GM-CSF expression and 43% of cellular M-CSF expression. The colocalization of CD90 and GM-CSF and CD90 and M-CSF was partly associated with the distribution of CD206^+^ and FRβ^+^ macrophages, respectively, in the adventitia (Fig. 2A). In addition, we observed abundant expression of CD90, GM-CSF and CD206 within the granuloma (Supplementary Fig. S2F). In GCA-positive aorta tissues, CD90, CD68, M-CSF, CD206 and FRβ were detected in structurally disrupted media, whereas GM-CSF expression was minimal. Approximately 30% of M-CSF was expressed by CD90^+^ cells. Limited colocalization of CD90 and M-CSF was observed at the site of CD68^+^ macrophages surrounding the necrotic areas in the media but not specifically in the areas with abundant FRβ^+^ macrophages (Fig. 2B).

### Macrophage-derived cytokines skew adventitial fibroblasts towards a pro-inflammatory and pro-destructive phenotype *in vitro*

**Figure 3.**
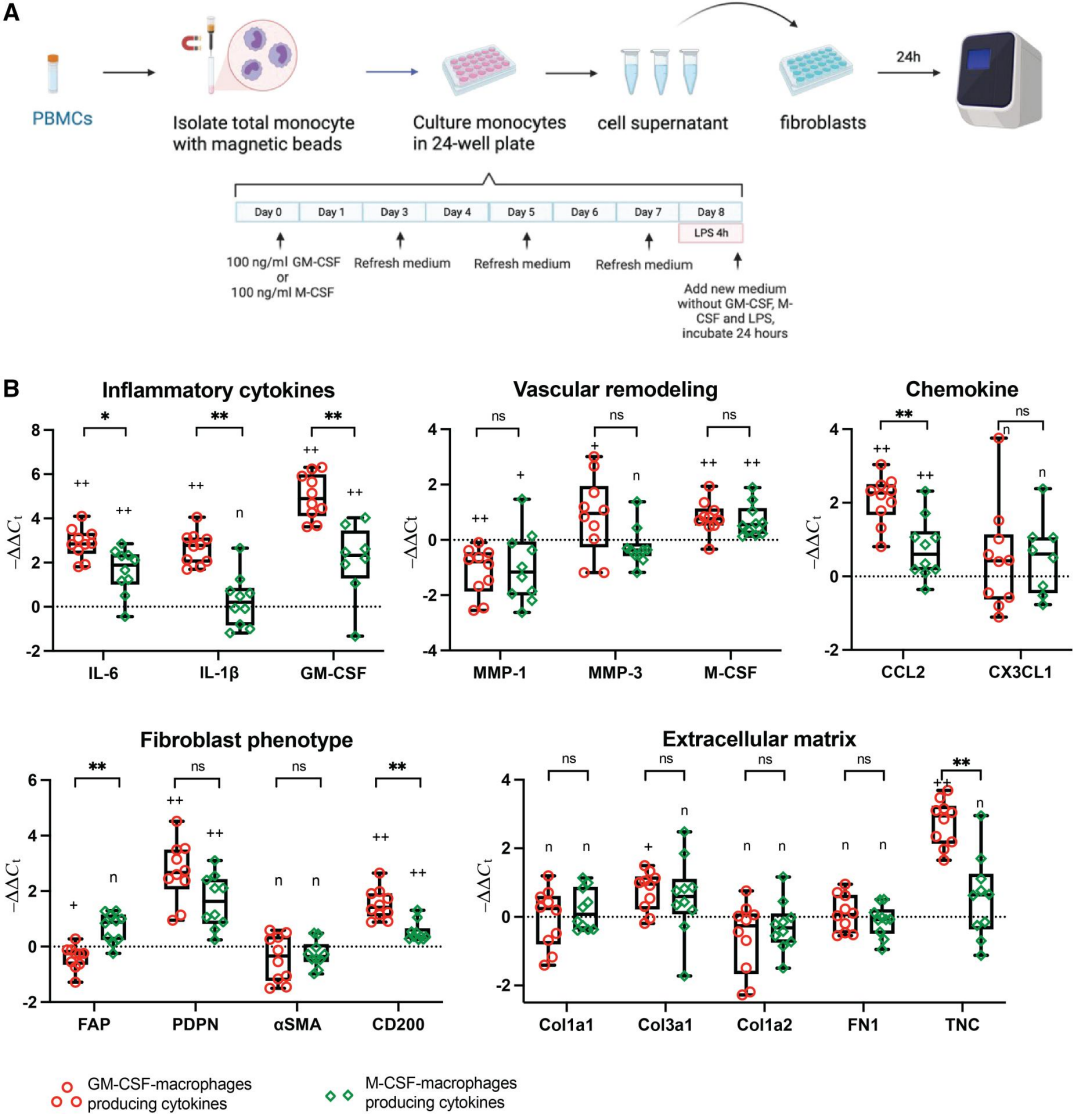
The impact of GM-CSF and M-CSF-derived macrophage conditioned medium on primary adventitial fibroblasts *in vitro*. (**A**) Experimental design: monocytes isolation, differentiation and activation; macrophage-conditioned medium was collected for fibroblast culture. (**B**) mRNA expression of pro-inflammatory cytokines (IL-6, IL-1β), monocyte/macrophages related growth factors (GM-CSF, M-CSF) and chemokines (CCL2, CX3CL1), matrix metalloproteinase (MMP-1, MMP-3), fibroblast phenotype markers (fibroblast activation protein [FAP], podoplanin [PDPN], α-smooth muscle actin [α-SMA], CD200), extracellular matrix production (type I collagen α 1 [Col1a1], type I collagen α 2 [Col1a2], type 3 collagen α 1 [Col3a1], fibronectin-1 [FN1] and tenascin-C [TNC]) in fibroblasts. Data are presented as median (interquartile range); +*P* < 0.05, ++*P* < 0.01, n: not significant compared with control (normal fibroblast culture); *P < 0.05, **P < 0.01, ns: not significant between the impact of GM-CSF macrophages and M-CSF macrophages on fibroblasts

Previously, we described two important macrophage subtypes in GCA lesions: CD206^+^ macrophages and FRβ^+^ macrophages, steered by GM-CSF and M-CSF, respectively [11]. To assess the impact of macrophage-derived soluble factors on fibroblasts, monocytes isolated from PBMCs were cultured with GM-CSF or M-CSF to differentiate into macrophages, and macrophage conditioned medium (MCM) was collected (Fig. 3A). As expected, GM-CSF-derived macrophages exhibited a more pro-inflammatory profile, as evidenced by the higher levels of IL-6, GM-CSF, TNF-α and IL-1β in MCM compared with M-CSF-derived macrophages, which demonstrated higher levels of IL-10 (Supplementary Fig. S3). GM-CSF- and, albeit to a lesser extent, M-CSF-derived macrophages upregulated mRNA expression of IL-6, GM-CSF, M-CSF, CCL-2, podoplanin (PDPN) and CD200 and downregulated mRNA expression of MMP-1 in cultured fibroblasts. Upregulation of IL-1β, MMP-3, Col3a1 and tenascin-C in fibroblasts was observed only in GM-CSF-derived macrophages group (Fig. 3B, Supplementary Fig. S4). FAP expression was only downregulated by the GM-CSF-derived macrophage supernatant. Both types of MCM showed no significant effects on mRNA expression of α-smooth muscle actin (α-SMA), Col1a1, Col1a2, fibronectin-1 and CX3CL1 in fibroblasts (Fig. 3B, Supplementary Fig. S4).

### Fibroblasts express MMP-3 and tenascin-C at the GCA vascular lesion

**Figure 4.**
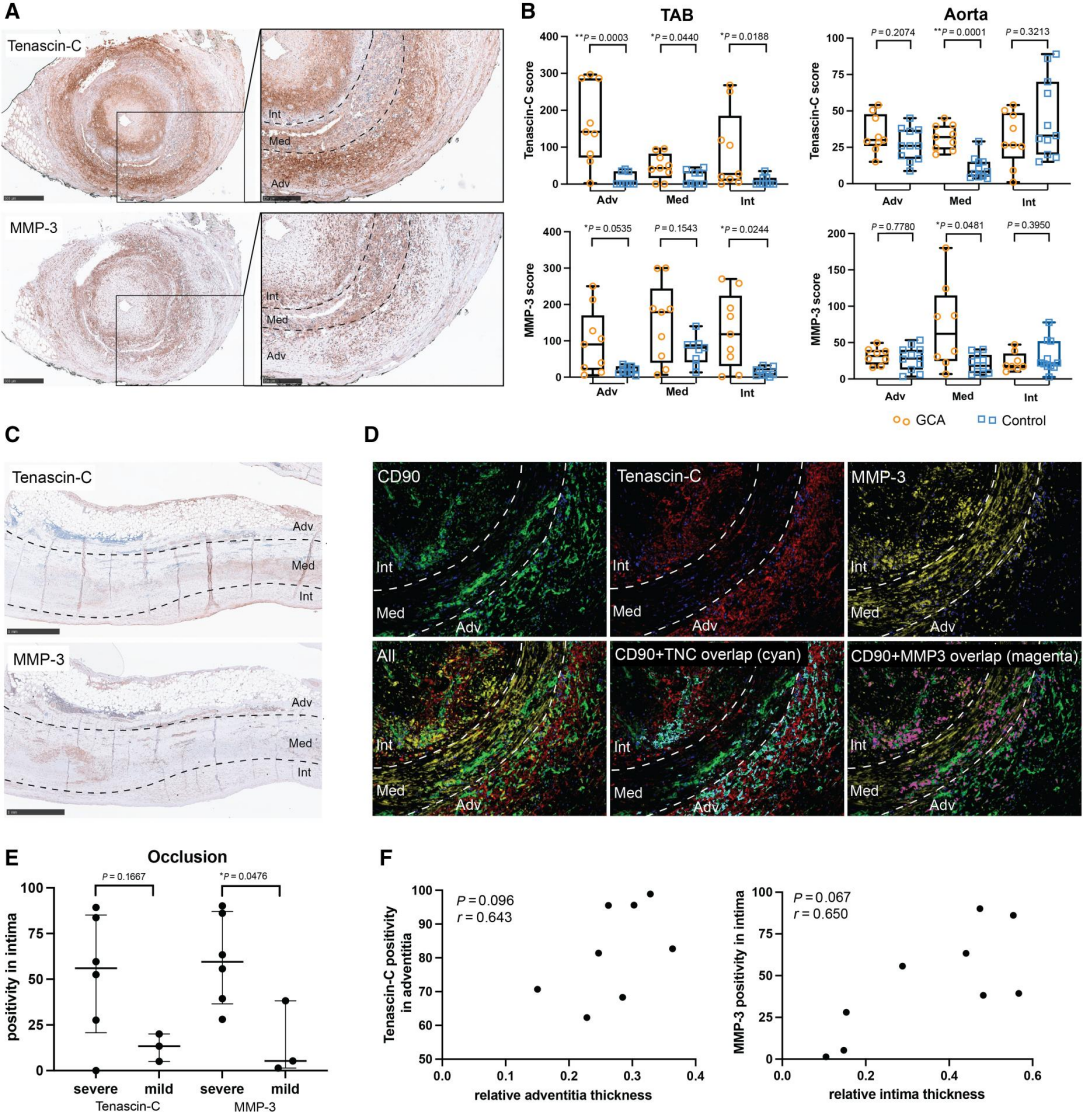
Local expression of tenascin-C and MMP-3 in inflamed GCA arteries. (**A**) IHC staining of tenascin-C and MMP-3 in GCA-positive temporal arteries. (**B**) IHC score of tenascin-C and MMP-3 in temporal artery biopsy (TAB) and aorta tissues calculated as percentage of positive cells × relative intensity. (**C**) IHC staining of tenascin-C and MMP-3 in GCA-affected aorta tissues. (**D**) Opal staining of CD90 (green), MMP-3 (yellow) and tenascin-C (red) in inflamed GCA TAB. Overlap of CD90 and tenascin-C and of CD90 and MMP-3 staining is shown in cyan and magenta, respectively. (**E**) High MMP-3 positivity in the intima is associated with severe occlusion of the GCA-affected TAB. (**F**) Intimal MMP-3 and adventitial tenascin-C positivity are not correlated with intimal and adventitial hyperplasia, respectively. Data are presented as median (interquartile range); ns: not significant, *P < 0.05, **P < 0.01. Adv: adventitia; Int: intima; Med: media

MMP-3 and tenascin-C are key contributors to tissue damage in various diseases [15, 20, 24]. In our *in vitro* experiments, tenascin-C was strongly upregulated by GM-CSF-derived macrophage supernatant only, whereas MMP-3 expression was slightly increased at the group level, albeit with high variance. To assess the presence of tenascin-C and MMP3 in TAB and aorta tissues, immunohistochemical staining was performed. In TAB, tenascin-C expression was significantly elevated across all three layers of GCA-affected tissues compared with controls, while MMP-3 showed a highly variable expression, which showed a statistically significant increase in the adventitia and intima of GCA-affected TAB (Fig. 4A and B, Supplementary Fig. S5A). Aorta tissues exhibited a distinct pattern than observed in temporal artery tissues. Tenascin-C and MMP-3 were both elevated in the media of the GCA-affected aorta compared with the atherosclerotic aorta, although overall, tenascin-C expression was much weaker than in TAB (Fig. 4B and C, Supplementary Fig. S5B and C). We also studied whether fibroblasts expressed MMP-3 and tenascin-C in GCA-affected TAB by Opal staining (Fig. 4D): a substantial overlap between CD90 and tenascin-C was observed in the adventitia, with CD90^+^ cells accounting for 90% of adventitial tenascin-C expression; CD90^+^ cells contributed to ∼30% of overall MMP-3 expression. We next investigated the relationship between tenascin-C/MMP-3 and vascular hyperplasia. TABs with severe intimal hyperplasia exhibited higher MMP-3 positivity than those with mild intimal hyperplasia (Fig. 4E). However, MMP-3 expression was not correlated with the degree of intimal hyperplasia (*P* = 0.067, *r* = 0.650) and adventitial tenascin-C positivity was not correlated with adventitial thickness (*P* = 0.096, *r* = 0.643) (Fig. 4F).

### Serum tenascin-C levels are significantly elevated in patients with GCA at diagnosis, and also in treatment-free remission

**Figure 5.**
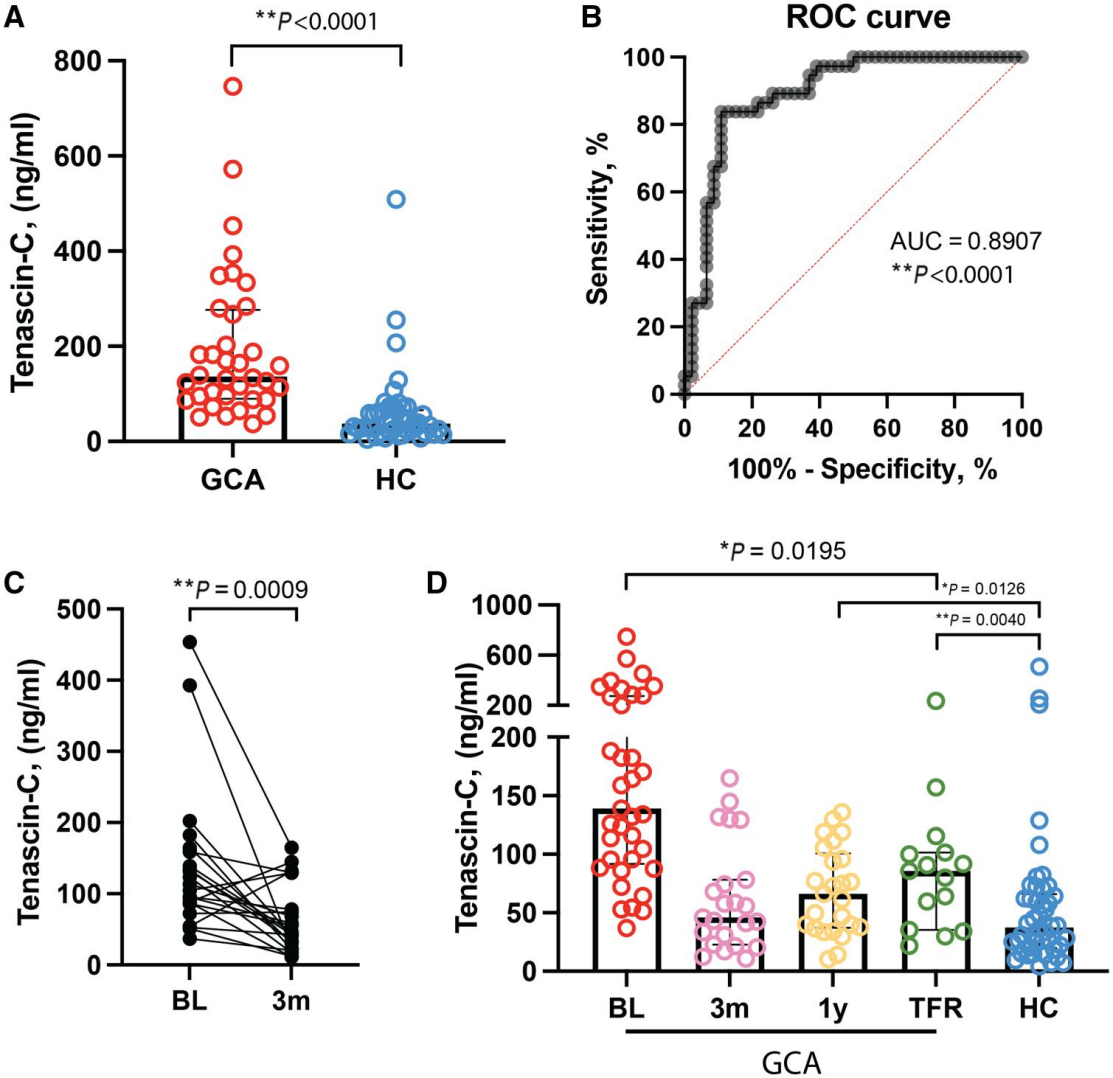
Serum tenascin-C levels in patients with giant cell arteritis (GCA) at baseline and at follow-up. (**A**) Treatment-naïve GCA patients showed higher serum tenascin-C levels than healthy controls. (**B**) Receiver operating characteristic (ROC) curve and area under the curve (AUC) score showing ability of tenascin-C to distinguish GCA patients from healthy controls. (**C**) Tenascin-C levels in GCA patients at 3 months (3m) *vs* baseline (BL). (**D**) An overview of serum tenascin-C levels in GCA patients at baseline, 3 months, 1 year (1y), treatment-free remission (TFR) and in healthy controls. Data are presented as median (interquartile range). ns: not significant, *P < 0.05, **P < 0.01

At baseline, serum tenascin-C levels were significantly higher in GCA patients than in healthy controls (median [interquartile range]: 136.5 [186.6] *vs* 37.5 [46.17] ng/ml) (Fig. 5A). The AUC of the ROC curve was 0.8907 (95% CI: 0.8191, 0.9623, with a positive likelihood ratio of 3.4, and negative likelihood ratio of 0.15), indicating good diagnostic accuracy for distinguishing patients with active GCA from controls (Fig. 5B). Baseline tenascin-C levels did not correlate with ESR, CRP, FAP, monocyte count and age (Supplementary Fig. S6A). Subgroup analysis based on GCA symptoms (C-GCA/LV-GCA/combination), PMR overlap, presence of (jaw and limb) claudication, and sex showed no significant differences (Supplementary Fig. S6B). At 3 months, tenascin-C levels were significantly decreased compared with baseline in GCA and they did not differ from HC (Fig. 5C and D). Tenascin-C levels were inversely correlated with cumulative glucocorticoid (GC) dose at 3 months but not with GC dose at 1 year (Supplementary Fig. S6C and D). Tenascin-C levels at 1 year and in treatment-free remission (TFR) remained lower than baseline GCA levels; however, they were higher than the levels in HC (Fig. 5D).

## Discussion

In this study, we provide evidence for crosstalk between vascular fibroblasts and macrophages in persistent inflammation and remodelling of GCA. In GCA-affected arteries, pro-inflammatory CD90^+^IL-6^+^ fibroblasts, rather than pro-resolving CD90^+^CD200^+^ fibroblasts, are spatially associated with macrophages. Vascular fibroblasts represent an important source of GM-CSF and M-CSF, which are associated with macrophage differentiation and subset distribution in GCA arterial lesions. *In vitro*, GM-CSF-derived macrophage supernatant, and to a lesser extent M-CSF-derived macrophage supernatant, skew fibroblasts towards a pro-inflammatory and pro-destructive phenotype. Arterial fibroblasts in GCA vessels produce MMP-3 and tenascin-C, which likely contribute to tissue damage. Serum tenascin-C should be investigated further as a biomarker for GCA diagnosis.

In GCA-affected arteries, different fibroblast subsets were spatially associated with macrophages. We observed weak CD200 expression at sites of inflammation in GCA-affected TAB and aortic tissues. This aligns with previous findings in RA synovium, where CD200 expression was inversely correlated with disease activity, as measured by DAS28 [15]. In RA synovium, MMP-3^+^/IL-6^+^ fibroblasts were found to colocalize with pro-inflammatory immune cells in regions with active inflammation, whereas CD200^+^/DKK3^+^ fibroblasts colocalized with pro-resolving immune cells (such as type 2 innate lymphoid cells and eosinophils) in regions without apparent inflammation [15]. Similarly, we observed that CD90^+^IL-6^+^ fibroblasts were located more in the vicinity of macrophages, whereas CD90^+^CD200^+^ fibroblasts were located more distantly from macrophages in GCA-affected TAB and aorta tissues. These findings indicate that a limited population of CD200 fibroblasts may contribute to, but are not sufficient for, the resolution of disease in GCA. In arthritis, synovial fibroblasts can acquire a more pro-resolving phenotype under treatment with disease-modifying anti-rheumatic drugs [15]. Importantly, the effect of IL-17A blockade was more pronounced than that of TNF-α blockade, on expression of both FAP (downregulation) and CD200 (upregulation) in arthritis. In that regard it would be interesting to investigate the effect of IL-17A inhibition, which showed promising effects in a recent phase 2 trial on remodelling and fibroblast phenotypes in GCA [25].

*In vitro*, GM-CSF-derived macrophage, and to a lesser extent M-CSF-derived macrophage conditioned supernatant, can skew fibroblasts towards a pro-inflammatory and pro-destructive phenotype. In RA, heparin binding epidermal growth factor (HBEGF)^+^ inflammatory macrophages were reported to be enriched in the inflamed synovial tissues and to promote fibroblast invasiveness in an EGF receptor-dependent manner [26]. Synovial tissue macrophages can elicit both inflammatory responses, characterized by increased expression of MMP1/3, RANKL, IL-6 and CCL2, among others, and repair responses, characterized by increased expression of collagen genes and TGF-β response genes, respectively, in primary fibroblast-like synoviocytes [27]. Moreover, skin fibroblasts from patients with SSc showed upregulated pathways associated with interferon responses and inflammation (IL-6, IL1-β, TNF, etc.) when cocultured with blood-derived human SSc macrophages [28]. In agreement with these studies, we found that macrophage-conditioned medium modulated phenotypic and functional features of human aortic adventitial fibroblasts. Upregulation of IL-6, IL-1β, GM-CSF and PDPN mRNA expression in fibroblasts indicates the promotion of a pro-inflammatory phenotype. Concurrently, the modulation of MMP-1, MMP-3, Col3a1 and tenascin-C expression suggests involvement in vascular damage and remodelling.

Our data show that fibroblasts can produce GM-CSF and M-CSF both in GCA vessels and *in vitro*. We previously reported that local production of GM-CSF and M-CSF was responsible for the skewing towards CD206^+^ macrophages and FRβ^+^ macrophages, respectively, in GCA lesions [11]. Here, we found that fibroblasts represent a major source of GM-CSF and M-CSF in the adventitia of GCA-positive TAB, which was closely related to the distribution of CD206^+^ macrophages and FRβ^+^ macrophages, respectively. We also showed *in vitro* that both GM-CSF-derived and M-CSF-derived macrophage products enhanced the mRNA expression of GM-CSF, M-CSF and CCL-2 in fibroblasts, indicating the potential impact of fibroblasts on monocyte/macrophage recruitment and differentiation. These results are in line with those of Kvedaraite *et al.* who found that intestinal PDGFRA^+^CD142^−/low^ fibroblasts fostered monocyte transition to CCR2^+^CD206^+^ macrophages through GM-CSF [29], and Stock *et al.* who reported cardiac fibroblasts as a major source of GM-CSF production in a mouse model of Kawasaki disease [30]. Recent studies have demonstrated that the GM-CSF receptor α blocker mavrilimumab is effective in reducing the incidence of flares in a clinical trial involving patients with GCA. Specifically, flares were observed in 8 out of 42 participants receiving mavrilimumab, compared with 13 out of 28 participants receiving a placebo [31]. In accordance, mavrilimumab reduced the expression of pro-inflammatory cytokines (IL-6, TNF-α, IL-1β) as well as molecules related to vascular injury (MMP-9, lipid peroxidation products and inducible nitric oxide synthase) in *ex vivo* cultured temporal arteries from GCA patients [32]. The finding that macrophage products induce GM-CSF in fibroblasts, which in turn drives macrophage differentiation, supports the therapeutic targeting of the GM-CSF pathway to halt disease chronicity in GCA by blocking macrophage–fibroblast crosstalk.

We show for the first time abundant tenascin-C expression in GCA TAB, primarily localized in adventitial fibroblasts. GM-CSF-derived macrophage products promoted the production of tenascin-C in vascular fibroblasts, suggesting that macrophage-fibroblast interactions might aggravate vascular damage. Moreover, elevated serum levels of tenascin-C in GCA patients highlights the potential of tenascin-C as a biomarker for GCA diagnosis. Increased serum tenascin-C levels have been previously reported in several other diseases, such as RA, SSc and cardiovascular diseases [22, 24, 33]. This indicates tenascin-C as a general marker for inflammation and damage rather than a specific marker for GCA. Serum tenascin-C levels at baseline were not correlated with inflammation parameters (ESR, CRP), which is consistent with a study in RA that reported no association with inflammation markers or DAS28, but positive correlation with joint erosion [22]. Although glucocorticoid downregulated serum tenascin-C levels significantly, tenascin-C levels were still higher at 1 year and in treatment-free remission compared with healthy controls. This is very similar to RA in which infliximab caused a transient drop in serum tenascin-C but it returned to increased levels at 1 year [22]. This long-term modulation of tenascin-C in chronic conditions differs from acute inflammation/injury whereby tenascin-C is rapidly but transiently induced. For instance, tenascin-C levels were significantly elevated in patients with acute myocardial infarction, while tissue tenascin-C expression peaked on days 3–5, and disappeared by day 28 in an experimental animal model of myocardial infarction [33]. Collectively, persistent elevation of tenascin-C during remission may reflect both ongoing tissue damage and subclinical inflammation in GCA.

Strengths of the current study are the combination of descriptive data in both temporal artery and aorta tissues, with blood measurements and *in vitro* mechanistic experiments. A limitation of this study is the use of PBMCs from HC (older than 50 years) rather than from treatment-naïve patients with GCA. This was based on previous studies that documented no disease-specific alterations of monocyte-derived macrophages [11, 34] in combination with limited availability of PBMCs from treatment-naïve patients with GCA. Future studies including monocytes from GCA patients, integrating coculture systems with single-cell RNA sequencing and spatial transcriptomics in GCA lesions will be essential to validate the crosstalk of macrophages and fibroblasts in GCA. Another limitation is the relatively small sample size for serum tenascin-C measurements at follow-up, which does not allow any conclusion regarding the value of tenascin-C as a predictor of disease relapse. The diagnostic and prognostic relevance of tenascin-C in GCA needs further validation in larger GCA cohorts across multiple follow-up time points, ideally including patients with advanced atherosclerosis as disease control.

In summary, the crosstalk between fibroblasts and macrophages not only enhances the production of proinflammatory cytokines, chemokines, MMPs and ECM-related proteins in fibroblasts, but may also facilitate monocyte recruitment and macrophage polarization, which contributes to persistent inflammation and abnormal remodelling of blood vessels. Our data provide support for therapeutic targeting of macrophage–fibroblast interactions in GCA.

## Supplementary material

Supplementary material is available at *Rheumatology* online.

## Supplementary Material

keaf408_Supplementary_Data

## Data Availability

Data are available upon reasonable request.
