# Supplementary material for: Crosstalk between macrophages and fibroblasts contributes to inflammation and damage in giant cell arteritis
Source: Rheumatology (Oxford). 2025 Aug 7;65(3):keaf408. doi: 10.1093/rheumatology/keaf408 (PMC13017568; doi:10.1093/rheumatology/keaf408)
Supplement: keaf408_Supplementary_Data [file keaf408_supplementary_data.zip › rhe-25-1071-File008.docx]

**Supplementary Tables**

**Supplementary Table S1. Antibodies used for immunohistochemistry (IHC) staining**

| Target | Antigen retrieval | Manufacturer & code | Host & isotype | Dilution | Secondary antibody | Dilution |
| --- | --- | --- | --- | --- | --- | --- |
| CD90 | pH 9 | NOVUS NBP2-37330 | Mouse IgG1 | 1:400 | DAKO Envision K4001 | undiluted |
| CD68 | pH 9 | DAKO M0876 | Mouse IgG3 | 1:50 | DAKO Envision K4001 | undiluted |
| Tenascin-C | pH 9 | Proteintech 67710-1-Ig | Mouse IgG1 | 1:650 | DAKO Envision K4001 | undiluted |
| MMP-3 | pH 9 | ABclonal, A11418 | Rabbit IgG | 1:100 | VisUcyte | undiluted |
| CD200 | pH 9 | ABclonal A21226 | Rabbit IgG | 1:100 | VisUcyte | undiluted |

**Supplementary Table S2. Antibodies used for immunofluorescence (IF) and OPAL staining**

| Target | Antigen retrieval | Manufacturer & code | Host & isotype | Dilution |
| --- | --- | --- | --- | --- |
| CD90 | pH 9 | NOVUS, NBP2-37330 | Mouse IgG1 | 1:300 |
| CD90 | pH 9 | Proteintech, 66766-1-Ig | Mouse IgG2a | 1:80 |
| CD68 | pH 9 | DAKO M0876 | Mouse IgG3 | 1:100 |
| IL-6 | pH 9 | Santa Cruz, sc-130326 | Mouse IgG2b | 1:25 |
| CD200 | pH 9 | ABclonal A21226 | Rabbit mono IgG | 1:100 |
| GM-CSF | pH 9 | Neobiotechnologies 1437-MSM3-P1ABX | Mouse IgG2c | 1:1000 |
| M-CSF | pH 9 | Abcam, ab52864 | Rabbit mono IgG | 1:100 |
| CD206 | pH 9 | R&D systems, MAB25341 | Mouse IgG2b | 1:50 |
| FR-β | pH 9 | Origene, TA808017 | Mouse IgG1 | 1:50 |
| MMP-3 | pH 9 | ABclonal, A11418 | Rabbit mono IgG | 1:100 |
| Tenascin-C | pH 9 | Proteintech 67710-1-Ig | Mouse IgG1 | 1:600 |

**Supplementary Table S3. Triple IF staining-CD90/FRβ/M-CSF**

|  | CD90 (Proteintech) | FRβ | M-CSF | Nucleus |
| --- | --- | --- | --- | --- |
| Primary ab | Mouse IgG2a, 1:75 | Mouse IgG1, 1:50 | Rabbit IgG, 1:100 |  |
| Secondary ab | Rat anti-mouse IgG2a (Biolegend, RMG2a-1)  1:40 | Goat anti-mouse IgG1 (Southern Biotech, 1071-01)  1:50 |  |  |
| Tertiary ab | Donkey anti-rat IgG  (abcam, ab150155)  1:50 | Donkey anti-goat IgG  (abcam, ab150129)  1:50 | Donkey anti-rabbit IgG  (abcam, ab175692)  1:50 |  |
| Conjugate/dye | AF647 | AF488 | AF568 | DAPI |

**Supplementary Table S4. OPAL staining patterns**

|  | OPAL480 | OPAL520 | OPAL570 | OPAL650 |
| --- | --- | --- | --- | --- |
| IL-6/CD90/CD68/CD200 | IL-6 1:25* | CD90 1:300 | CD68 1:100 | CD200 1:100* |
| CD90/CD68/IL-6 |  | CD90 1:300 | CD68 1:100 | IL-6 1:25 |
| CD90/CD68/CD200 |  | CD90 1:300 | CD68 1:100 | CD200 1:100* |
| CD90/MMP-3/Tenascin-C |  | CD90 1:300 | Tenascin-C 1:600 | MMP-3 1:100* |
| CD90/GM-CSF/CD206 |  | CD90 1:300 | CD206 1:50 | GM-CSF 1:1000 |

*Overnight incubation of primary antibody at 4°C.

**Supplementary Figures**

**
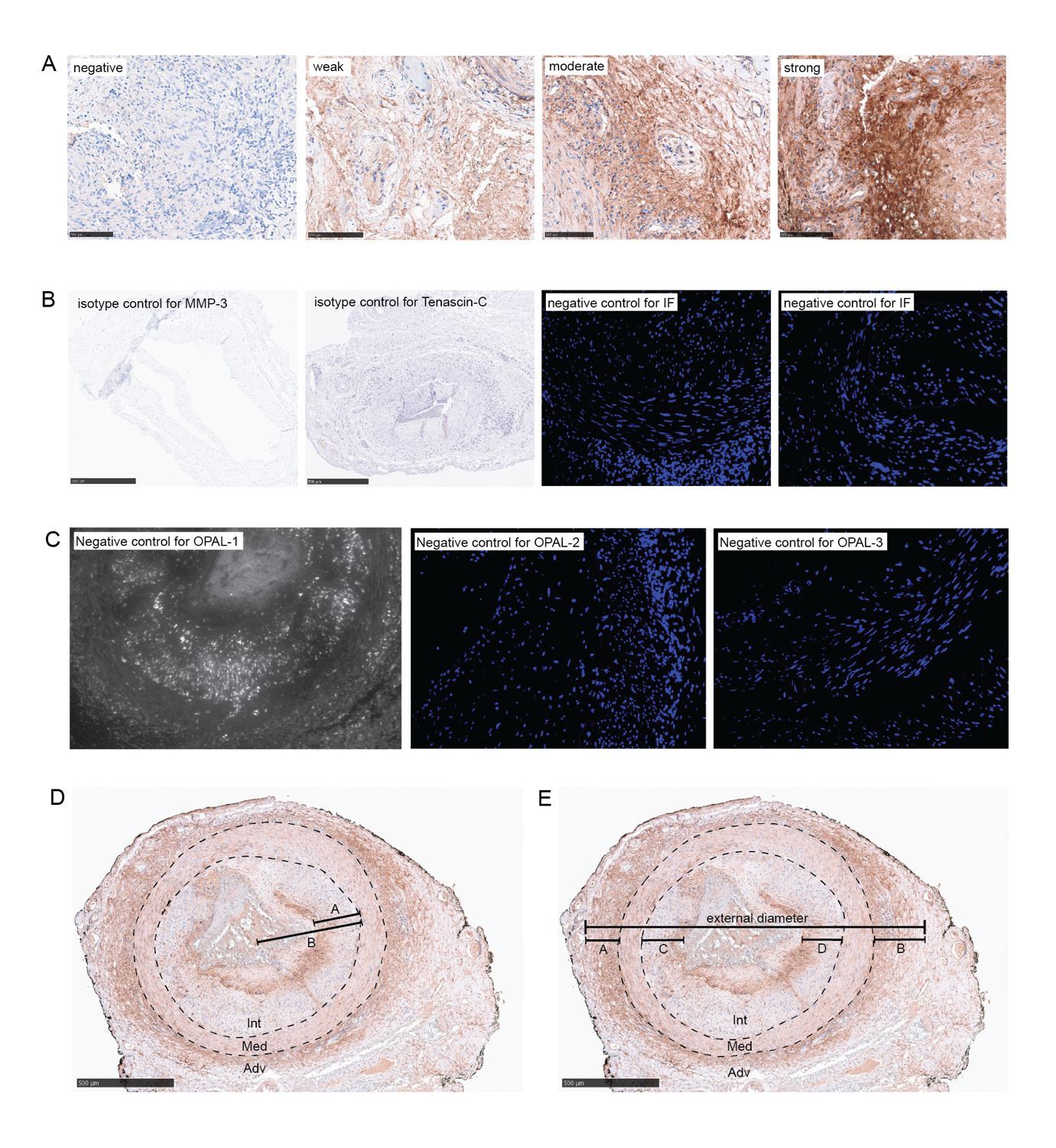
**

**Supplementary Figure S1. Control and score for staining.** (A) The relative intensity of immunohistochemistry (IHC) staining. Score: negative=0, weak staining=1, moderate staining=2, strong staining=3. (B) Isotype control for Tenascin-C and MMP-3 staining and negative control for immunofluorescence (IF) staining. (C) Isotype control for OPAL staining. 1=control for 4-color OPAL; 2=mouse-OPAL520, mouse-OPAL570, rabbit-OPAL650, 3=mouse-OPAL520, mouse-OPAL570, mouse-OPAL650. (D) Intima thickness score = A/B. (E) Relative adventitia thickness score = (A+B)/external diameter, relative intima thickness score = (C+D)/external diameter. Adv=adventitia, Med=media, Int=intima.


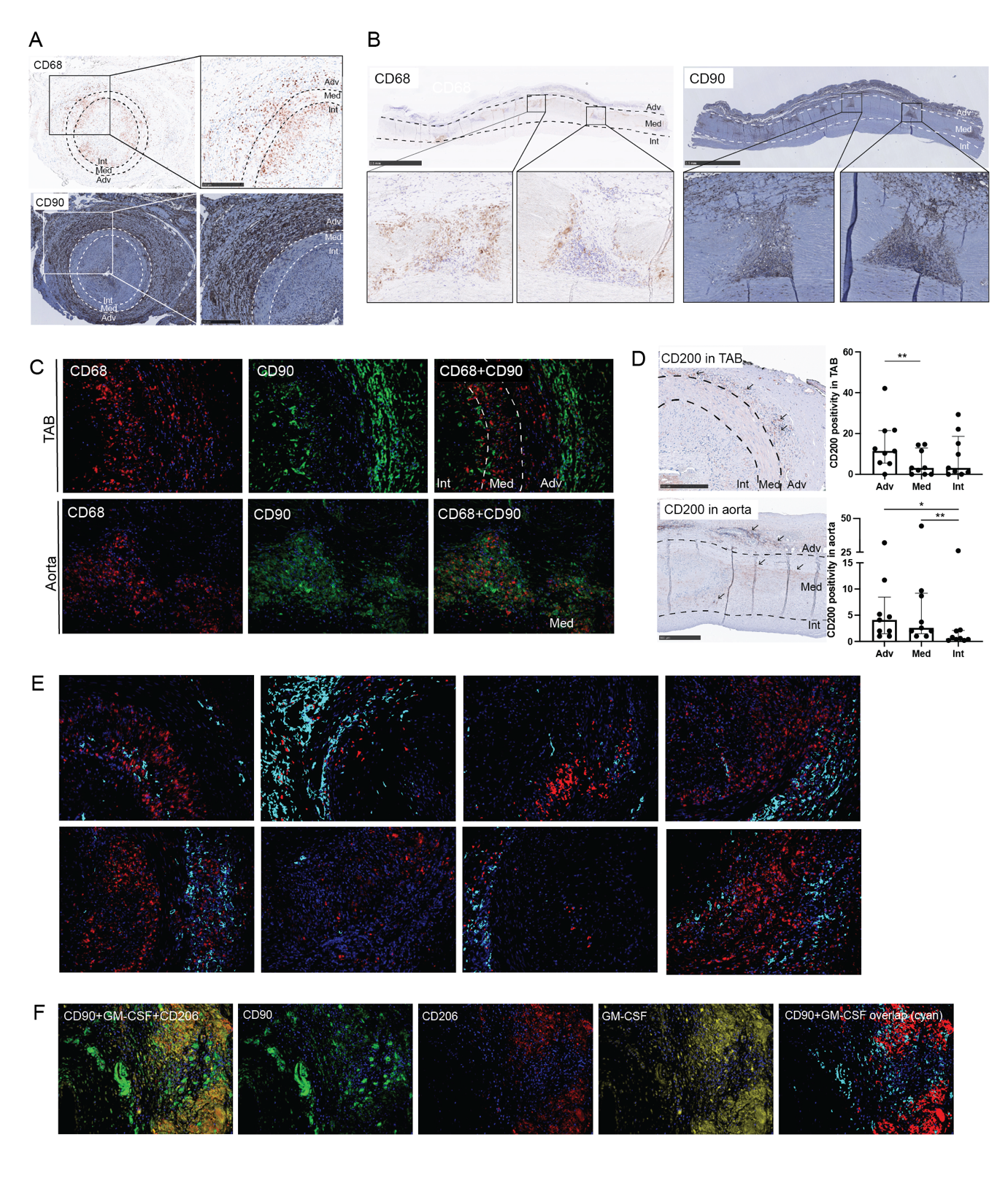


**Supplementary Figure S2. Detection of fibroblasts and macrophages in giant cell arteritis (GCA)-affected temporal artery biopsies (TABs) and aorta tissues.** (A) IHC staining of CD68 and CD90 in TAB; (B) IHC staining of CD68 and CD90 in aorta; (C) OPAL staining of CD68 and CD90; (D) IHC staining of CD200 in GCA-affected temporal artery biopsies (TAB) and aorta tissues; (E) OPAL staining of CD200/CD90/CD68 in 8 different TABs shows the location of CD200^+^fibroblasts and macrophages (n=8). Red indicates CD68^+^macrophages, and cyan indicates CD90^+^CD200^+^fibroblasts. Adv=adventitia, Med=media, Int=intima. (F) Representative images in granuloma (GCA-affected TAB, media was fully destroyed) showing the distribution of CD90 (green), GM-CSF (yellow) and CD206 (red). The last image shows the colocalization of CD90/GM-CSF (cyan).


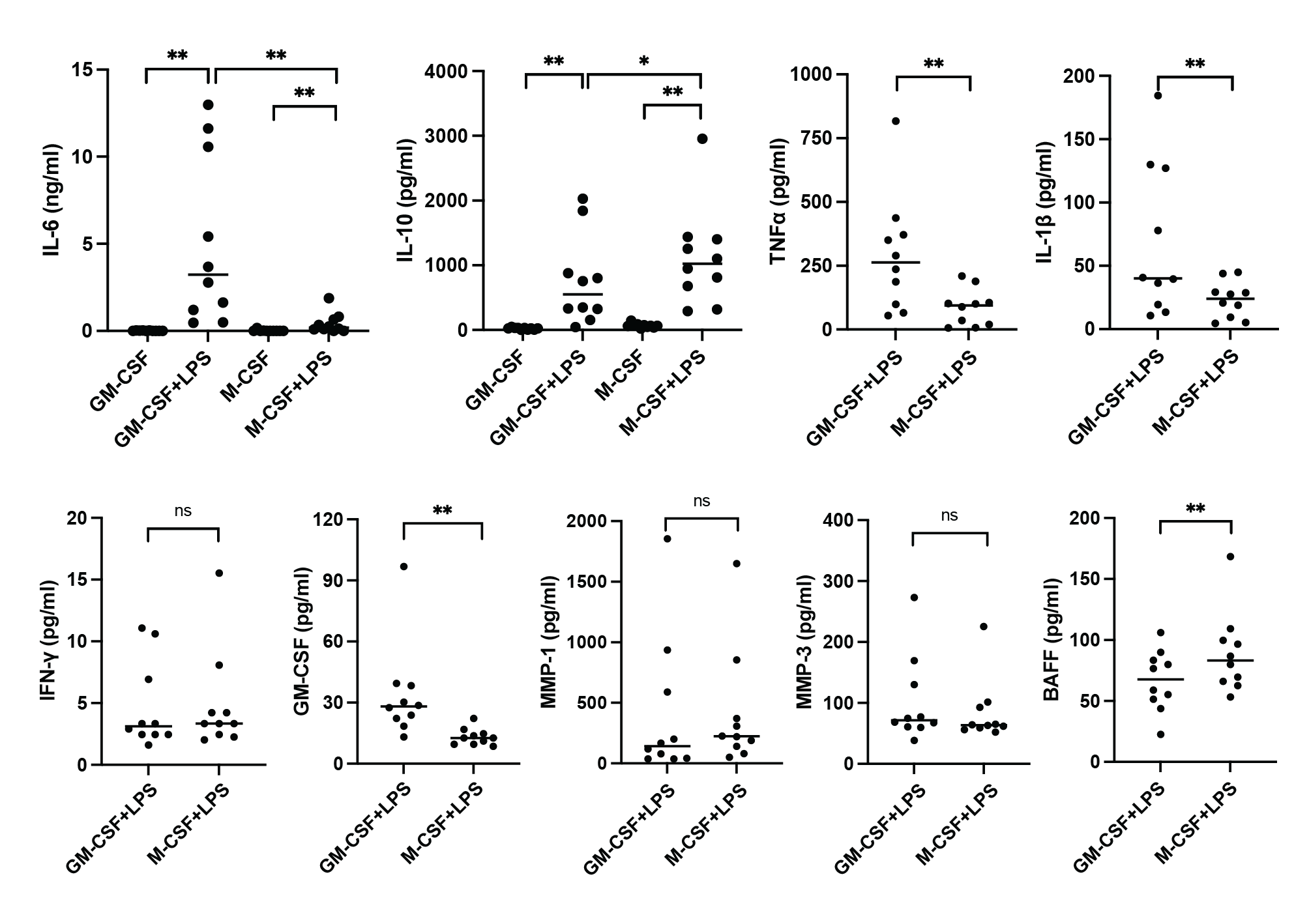


**Supplementary Figure S3. Cytokine detection in macrophage conditioned medium (MCM) from GM-CSF and M-CSF differentiated macrophage from healthy controls (n=10).** We first measured the production of pro-inflammatory cytokine interleukin (IL)-6 and anti-inflammatory cytokine IL-10 in MCM by ELISA, and observed significant higher levels of IL-6 and IL-10 in groups with LPS activation. Then we measured production of tumor necrosis factor alpha (TNF-α), IL-1β, interferon gamma (IFN-γ), granulocyte-macrophage colony-stimulating factor (GM-CSF), matrix metalloproteinase (MMP)-1, MMP-3, B-cell activating factor (BAFF) in activated groups by Luminex. *p<0.05, **p<0.01, ns=not significant.


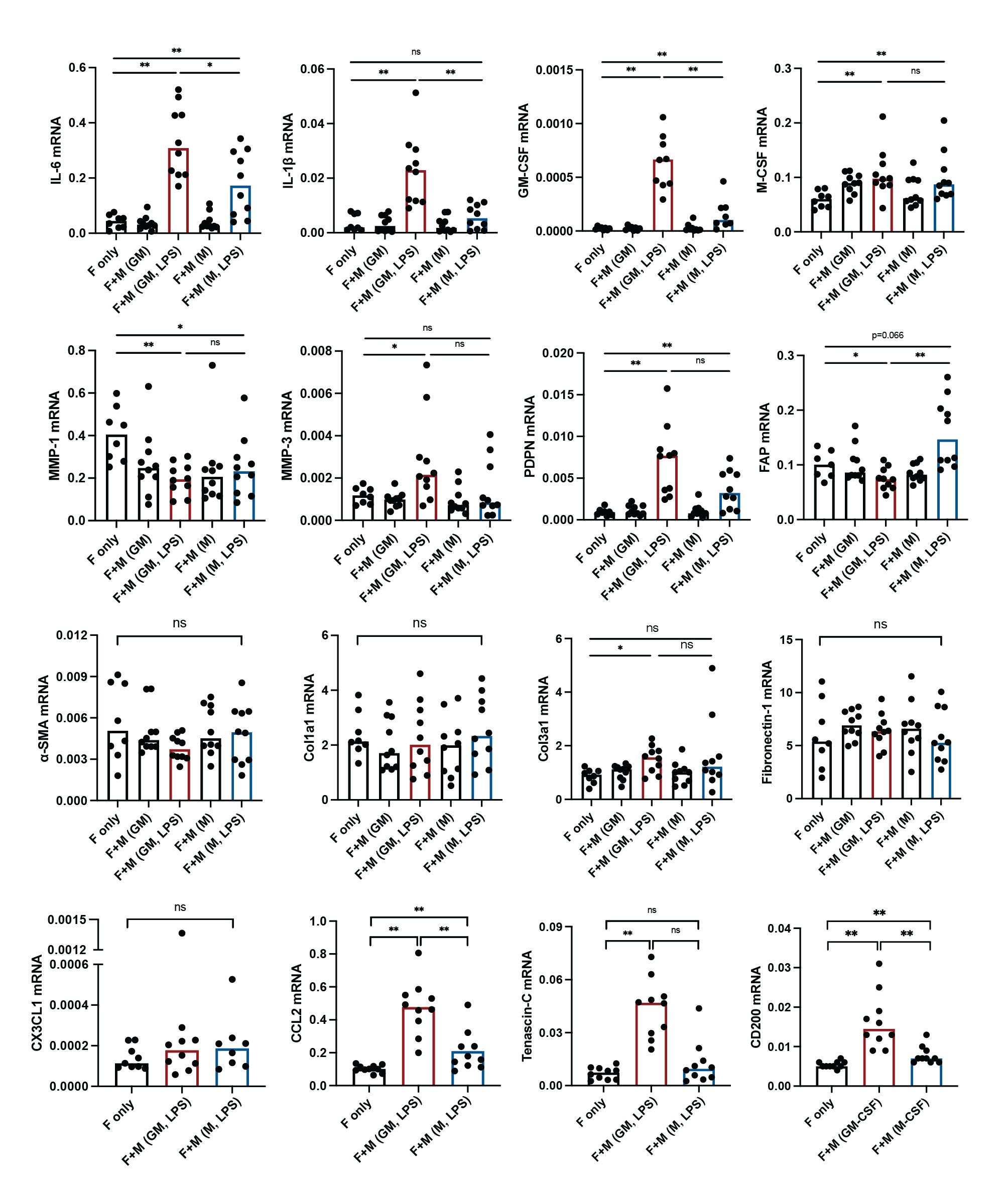


**Supplementary Figure S4. The impact of macrophage conditioned medium on fibroblasts.** mRNA levels of interleukin-6 (IL-6), interleukin-1 beta (IL-1β), granulocyte-macrophage colony-stimulating factor (GM-CSF), granulocyte-macrophage colony-stimulating factor (M-CSF), Chemokine C-C motif ligand 2 (CCL2), Chemokine C-X3-C motif ligand 1 (CX3CL1), matrix metalloproteinase 1 (MMP-1), matrix metalloproteinase 3 (MMP-3), fibroblast activation protein alpha (FAP), podoplanin (PDPN), alpha smooth muscle actin (α-SMA), type I collagen alpha 1 (Col1a1), type I collagen alpha 2 (Col1a2), type 3 collagen alpha 1 (Col3a1), fibronectin-1, Tenascin-C, CD200 were shown as relative expression (to GAPDH). *p<0.05, **p<0.01, ns=not significant. F=fibroblasts, M=macrophage conditioned medium.


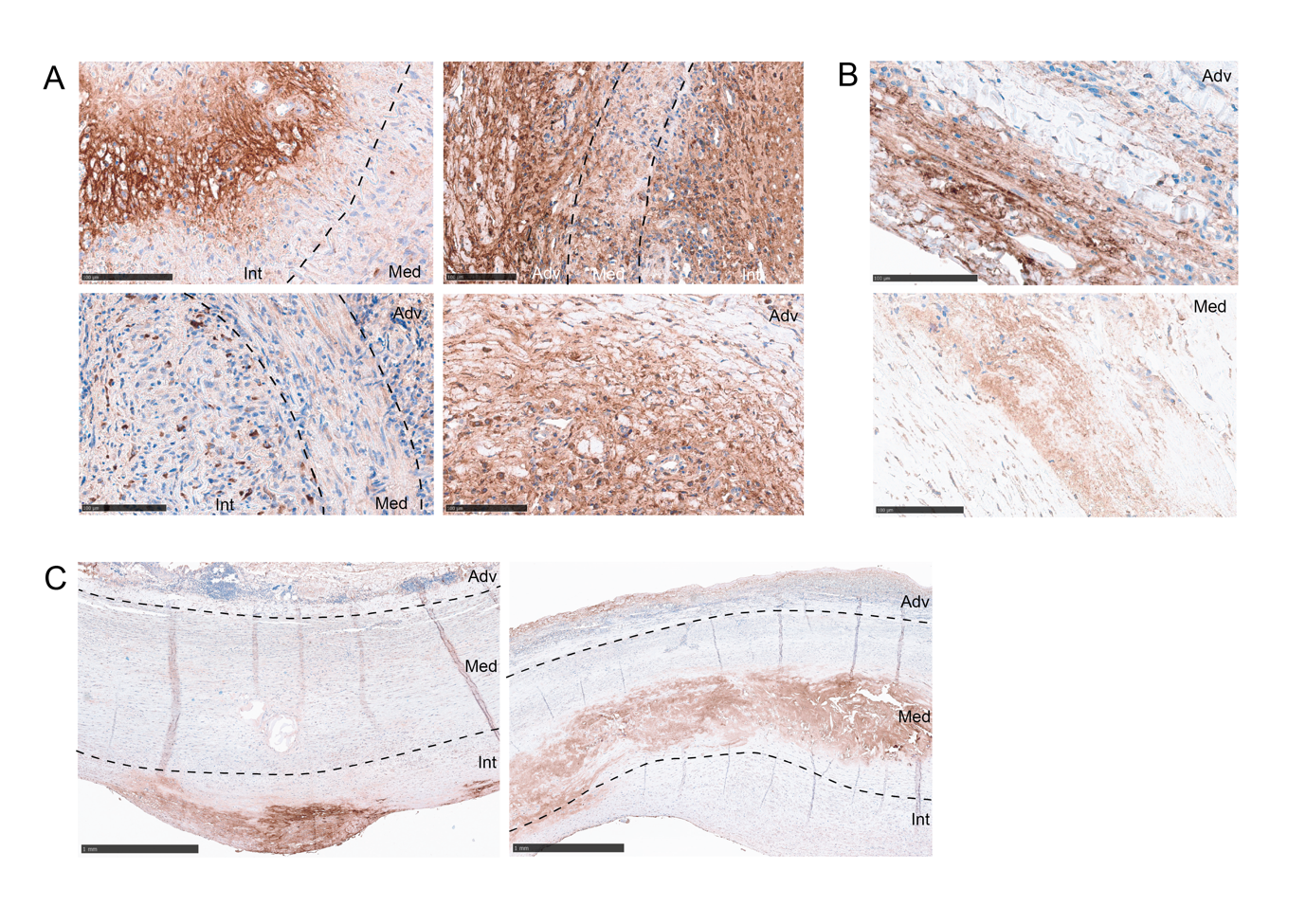


**Supplementary Figure S5. Additional data on tenascin-C tissue expression.** (A, B) Expression of tenascin-C in GCA-affected temporal arteries (A) and aorta tissues (B) at high magnification. (C) Expression of tenascin-C in atherosclerosis aorta. Adv=adventitia, Med=medium, Int=intima.


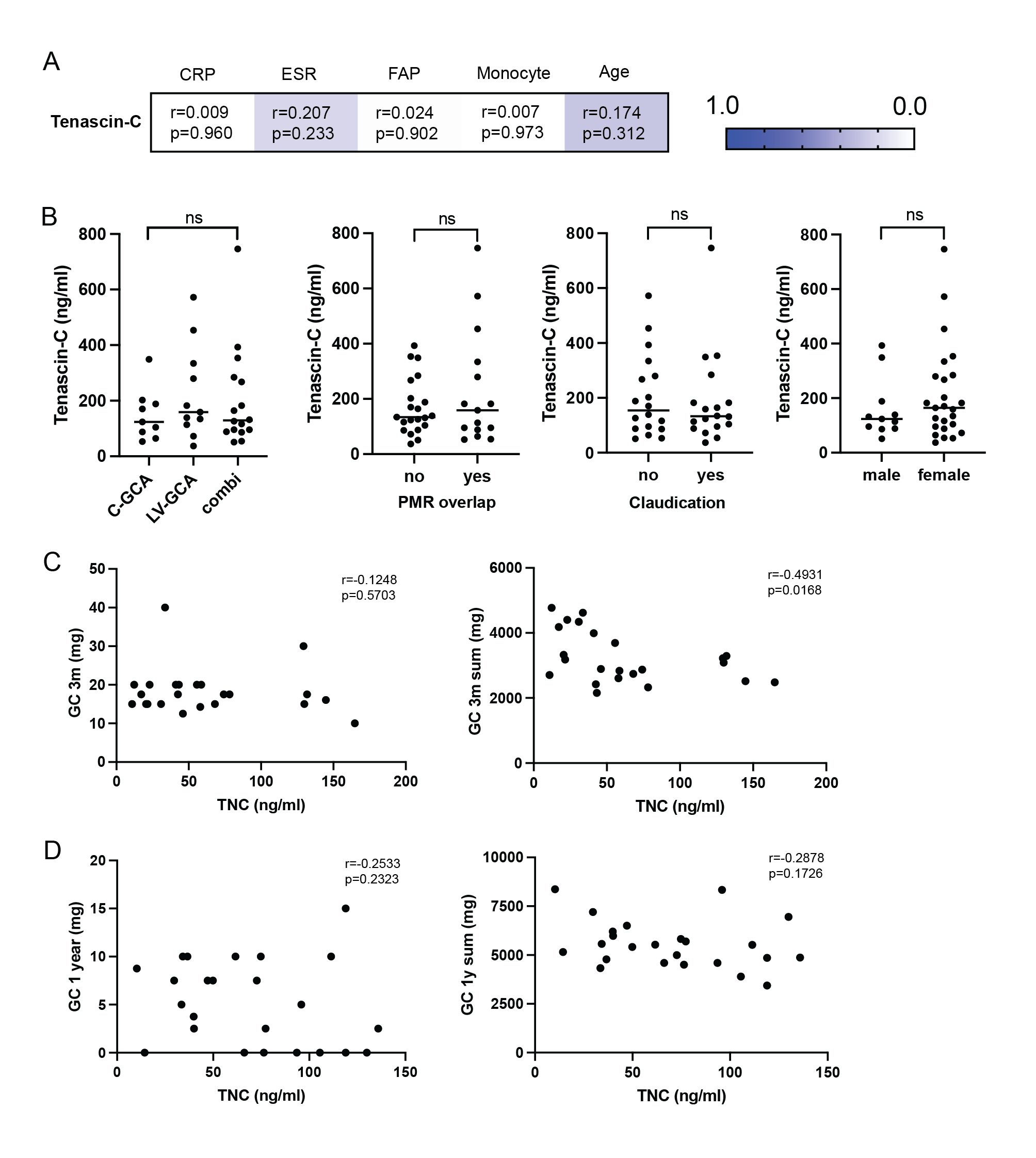


**Supplementary Figure S6. Serum Tenascin-C levels in patients with GCA in relation to their clinical features.** (A) No correlation between baseline Tenascin-C levels and CRP, ESR, FAP, monocyte count, age. (B) Subgroup analysis based on GCA symptoms (C-GCA/LV-GCA/combination), PMR overlap, presence of (jaw- and limb-) claudication, sex showed no significant differences. Ns=not significant. (C) Correlation of Tenascin-C levels with glucocorticoid dose (GC) at 3 months (3m). GC 3m sum is total GC dose for the first 3 months. (D) Correlation of Tenascin-C levels with glucocorticoid dose (GC) at 1 year. GC 1y sum is total GC dose for the first year.
