# Supplementary material for: Crosstalk between macrophages and fibroblasts contributes to inflammation and damage in giant cell arteritis
Source: Rheumatology (Oxford). 2025 Aug 7;65(3):keaf408. doi: 10.1093/rheumatology/keaf408 (PMC13017568; doi:10.1093/rheumatology/keaf408)
Supplement: keaf408_Supplementary_Data [file keaf408_supplementary_data.zip › rhe-25-1071-File007.docx]

**Supplementary Data S1. Supplementary Methods**

**Immunohistochemistry (IHC) staining and Immunofluorescence (IF) staining**

Three micrometer sections from formalin-fixed, paraffin-embedded aorta and TAB were deparaffinized and rehydrated, followed by antigen retrieval in a Tris-EDTA buffer (pH=9). For IHC staining, the tissues were incubated with primary antibodies detecting CD90, CD68, CD200, tenascin-C, and MMP-3 (Supplementary Table S1). After endogenous peroxidase blockade was performed, tissues were subsequently incubated with secondary antibody for 40 min, DAB for visualization and hematoxylin for counterstaining. For IF staining, the tissues were incubated with a cocktail of primary antibodies at 4°C overnight, followed by incubation with a cocktail of secondary antibodies and fluorescence-labelled tertiary antibodies (Supplementary Tables S2 and S3). Finally, the tissues were incubated with DAPI for 10 min to visualize the nuclei and sealed. Image cubes were captured at a magnification of 200× using the Nuance Multispectral Imaging System 3.0.1 (PerkinElmer, Waltham, MA, USA) with NuanceFX 3.0.1 software (440:460 for DAPI=blue, 490:530 for Alexa 488=green, 570:600 for Alexa 568=red, 710:720 for Alexa 647=yellow).

Three layers of the inflamed temporal arteries and aortic tissues (adventitia, media, intima) were scored for tenascin-C and MMP-3 expression. The final score was calculated as the percentage of positive cells multiplied by the relative intensity. The percentage of positive cells per layer was assessed and calculated using Qupath 0.3.0. The relative intensity was defined as absent=0, weak=1, moderate=2 and strong=3 (Supplementary Figure S1A)[1]. Isotype controls for IHC and negative controls for immunofluorescence are shown in Supplementary Figure S1B.

For inflamed GCA TAB, the intimal thickness score was calculated as intimal thickness divided by the distance from the center of the lumen to the inner border of the media. Based on intima thickness score, the degree of intimal hyperplasia was divided into severe occlusion (>0.7) and mild occlusion (<0.7) [2]. The intimal thickness score was calculated as the intimal thickness divided by the external diameter of the whole vessel [3], the adventitia thickness score was calculated as the adventitia thickness divided by the external diameter of the entire vessel [3] (Supplementary Figure S1C and S1D).

**OPAL staining**

Colocalization of CD90/CD200/IL-6/CD68, CD90/GM-CSF/CD206 and CD90/MMP-3/Tenascin-C was analyzed by OPAL staining. Briefly, paraffin-embedded sections were deparaffinized in xylene and rehydrated with graded ethanol, followed by antigen retrieval in a heated Tris-EDTA buffer (pH=9) bath. The tissues were then incubated with primary antibody, secondary HRP antibody, and OPAL. After washing with demi water, the cycle was repeated twice or three time, starting from antigen retrieval, incubation of primary antibody, secondary antibody and OPAL. The tissues were then incubated with 4',6-diamidino-2-phenylindole (DAPI) for 10 min. Image cubes of 3-color OPAL and 4-color OPAL were captured using the Nuance Multispectral Imaging System 3.0.1 (at a magnification of 200×) and Thunder Imaging system (Leica) (100× and 400×), respectively. The OPAL staining patterns are shown in Supplementary Table S4.

**PBMC isolation, monocyte isolation and macrophage differentiation**

PBMCs were isolated from healthy donors (n=10) using density gradient centrifugation using SepMate^TM^ (StemCell) tubes. Approximately 10 million of PBMCs in 1 mL of the final cell suspension was added per cryovial. Monocytes were isolated from 2 vials of thawed PBMCs using the EasySep Human Monocyte Enrichment Kit without CD16 depletion (StemCell #19058). Following isolation, monocytes were cultured in the presence of 100 ng/mL GM-CSF (Peprotech 300-03) or M-CSF (Peprotech 300-25) for 8 days. Medium plus factors were replaced on days 3 and 5, whereby the culture supernatant was spun down (300 × g for 5 min), and the cell pellet was resuspended in medium containing factors and subsequently added to the monocyte culture. On day 8, macrophages were activated with 100 ng/mL LPS for 4 h. After 4 h, the cells were washed twice with PBS and cultured in fresh medium for 24 h to allow for the release of soluble factors into the cell supernatant (Figure 3A). The cell supernatants were collected for subsequent fibroblast culture and cytokine measurements.

**Fibroblast culture**

Human aortic adventitial fibroblasts (HAoAF) were obtained from PromoCell (C-12380), originating from the thoracic aorta of a 50-year-old Caucasian male donor. Cells were routinely cultured in low-glucose DMEM supplemented with 10% FCS, 50 μg/ml gentamycin, 10 μg/ml insulin-transferrin-selenium-sodium pyruvate (ITS-A), 1 ng/ml basic fibroblast growth factor (bFGF), and 500 μM Vit-C and were grown at 37 °C in a 5% CO_2_ humidified chamber. Cells used for all experiments were between passages 4 and 7.

**Fibroblasts cultured in macrophage supernatant**

HAoAF cells were seeded onto 24-well plates at a density of 25000/cm^2^ to reach 50% confluence after 24 h. The macrophage supernatant was mixed with fibroblast culture medium in a 1:1 ratio and added to the fibroblast culture. After 24 h, fibroblasts were collected for further analysis.

**Real-time quantitative PCR (qPCR)**

Total RNA was isolated from cultured fibroblasts using TRIzol™ and complementary DNA (cDNA) was obtained by reverse transcription using the PrimeScript RT Master Mix Kit (Invitrogen). qPCR was performed using the Absolute QPCR ROX mix on a ViiA7 machine. Relative standard curve method (2^-∆CT^) was used to determine the relative mRNA expression with probes targeting IL-6(Hs00174131_m1), IL-1β(Hs01555410_m1), GM-CSF(Hs00929873_m1), M-CSF(Hs00174164_m1), PDPN(Hs00366766_m1), FAP(Hs00990791_m1), α-SMA(Hs00909449_m1), CD200(Hs01033302_m1), MMP-1(Hs00899658_m1), MMP-3(Hs00968305_m1), CCL2(Hs00234140_m1), CX3CL1(Hs00171086_m1), Col1a1(Hs00164004_m1), Col1a2(Hs01028956_m1), Col3a1(Hs00943809_m1), fibronectin 1(Hs01549976_m1), tenascin-C(Hs01115665_m1). Gene expression was normalized to the expression of GAPDH (Hs99999905_m1). The -∆∆CT method was employed to compare the changes between the treatment group, consisting of fibroblast cultures with macrophage supernatant, and the control group, where fibroblasts were cultured in normal medium, which was set as 0.

**Enzyme-linked immunosorbent assay (ELISA) and Luminex**

Macrophage supernatants were analysed for IL-6 and IL-10 using the Human IL-6 DuoSet ELISA (DY206, R&D systems™) and Human IL-10 DuoSet ELISA (DY217B, R&D Systems ™) according to the manufacturer’s instructions. 40-fold and 2-fold diluted samples were measured in duplicate in a BioTek EPOCH 2 microplate reader for IL-6 and IL-10, respectively. The mean was calculated for the two measurements (standard curve range 0-600 pg/ml for IL-6, 0-2000 pg/ml for IL-10). In addition, activated macrophage supernatants (2-fold diluted) were analyzed using a human premix magnetic Luminex screen assay kit (R&D Systems) to detect GM-CSF, tumour necrosis factor alpha (TNF-α), IL-1β, interferon gamma (IFN-γ), MMP-1, MMP-3, B-cell activating factor (BAFF), and lymphotoxin alpha (LT-α). The assay was read on a Luminex Magpix instrument and analysed using xPONENT 4.2 software. Serum samples were analysed for tenascin-C levels using ELISA kits (ABclonal, RK12297, standard curve range 0-2000 pg/ml), according to the manufacturer’s instructions. 500-fold diluted samples were measured using a BioTek EPOCH 2 microplate reader.

Reference

1. Xu S, Jiemy WF, Boots AMH, Arends S, van Sleen Y, Nienhuis PH*, et al*. Altered Plasma Levels and Tissue Expression of Fibroblast Activation Protein Alpha in Giant Cell Arteritis. *Arthritis Care Res (Hoboken)* 2024; 76(9):1322-1332.

2. van Sleen Y, Jiemy WF, Pringle S, van der Geest KSM, Abdulahad WH, Sandovici M*, et al*. A Distinct Macrophage Subset Mediating Tissue Destruction and Neovascularization in Giant Cell Arteritis: Implication of the YKL-40/Interleukin-13 Receptor alpha2 Axis. *Arthritis Rheumatol* 2021; 73(12):2327-2337.

3. Kinoshita Y, Ishii H, Kushima H, Johkoh T, Yabuuchi H, Fujita M*, et al*. Remodeling of the pulmonary artery in idiopathic pleuroparenchymal fibroelastosis. *Sci Rep* 2020; 10(1):306.
